# Production of highly cytotoxic and low immunogenic L-asparaginase from *Stenotrophomonas maltophilia* EMCC2297

**DOI:** 10.1186/s13568-024-01700-9

**Published:** 2024-05-04

**Authors:** Nada A. Abdelrazek, Sarra E. Saleh, Marwa M. Raafat, Amal E. Ali, Mohammad M. Aboulwafa

**Affiliations:** 1 Department of Microbiology and Immunology, Faculty of Pharmacy, Ain Shams University, Al Khalifa Al Maamoun St., Abbassia, Cairo, 11517 Egypt; 2 Department of Microbiology and immunology, Faculty of Pharmacy, Future University in Egypt, Cairo, Egypt; 3 Department of Microbiology and Immunology, Faculty of Pharmacy, King Salman International University, South Sinai, Ras-Sudr, Egypt

**Keywords:** *Stenotrophomonas maltophilia*, L-asparaginase, Purification, Cytotoxicity, Immunogenicity

## Abstract

L-asparaginase is an important therapeutic enzyme that is frequently utilized in the chemotherapy regimens of adults as well as pediatric patients with acute lymphoblastic leukemia. However, a high rate of hypersensitivity with prolonged use has limited its utilization. *Stenotrophomonas maltophilia* (*S. maltophilia*) EMCC2297 isolate was reported as a novel and promising source for L- asparaginase. The present study aimed at the production, purification, and characterization of L- asparaginase from *S. maltophilia* EMCC2297 isolate. The microbial production of L-asparaginase by the test isolate could be increased by pre-exposure to chloramphenicol at 200 µg/ml concentration. *S. maltophilia* EMCC2297 L-asparaginase could be purified to homogeneity by ammonium sulphate precipitation and the purified form obtained by gel exclusion chromatography showed total activity of 96.4375 IU/ml and specific activity of 36.251 IU/mg protein. SDS-PAGE analysis revealed that the purified form of the enzyme is separated at an apparent molecular weight of 17 KDa. Michaelis-Menten constant analysis showed a Km value of 4.16 × 10^− 2^ M with L-asparagine as substrate and Vmax of 10.67 IU/ml. The antitumor activity of the purified enzyme was evaluated on different cell lines and revealed low IC50 of 2.2 IU/ml and 2.83 IU/ml for Hepatocellular cancer cell line (HepG-2), human leukemia cancer cell line (K-562), respectively whereas no cytotoxic effect could be detected on normal human lung fibroblast cells (MRC-5). However, mice treated with native L-asparaginase showed lower IgG titre compared to commercial L-asparaginase. This study highlights the promising characteristics of this enzyme making it a valuable candidate for further research and development to be an adduct in cancer chemotherapy.

## Introduction

Enzymes are unique in that, in addition to serving as therapy targets, they can also be used as therapeutic molecules (Vitolo 2009). Microbial enzymes have a number of benefits over enzymes from animals and plants. One of the most important benefits is that the microbial enzymes are more active and stable. L- asparaginase from *Erwinia carotovora* or *Escherichia coli* can be utilized in the management of acute lymphocytic leukemia (Eden et al. 1990). The enzyme specific activity on tumor cells arises from the fact that these cells lack asparagine synthetase enzyme, which is normally present in normal cells, that is responsible for the synthesis of amino acid L-asparagine from ammonia and aspartate (Gurung et al. 2013). Thus, by injecting L- asparaginase the availability of the amino-acid is decreased leading to destruction of the leukemic cells (Zhou et al. 2023).

Another application of L-asparaginase is in the food industry; One step towards preventing the overabundance of acrylamide synthesis in potato- and cereal-based foods is to apply L-asparaginase enzyme before heat treatment. It was effectively employed in cereal processing and the manufacturing of French fries and potato crisps, reducing acrylamide with 62% efficacy. Verified in bread, biscuits, gingerbread, and fried-dough pastry manufacturing, it exhibited a reduction of > 90% in acrylamide (Ciesarová and Kukurová 2024). Additionally, L-asparaginase, as a biosensor, can be used to measure L-asparagine content in both food industry and leukemic patients (Nunes et al. 2021).

L-asparaginases from bacterial sources have the advantages of being extracted and purified easily, thus can be produced on large scale (Shafqat et al. 2023). Accordingly, the present study focused on L-asparaginase as a safe and potential chemotherapeutic agent from a novel microorganism. *S. maltophilia* EMCC2297 L- asparaginase was produced, purified, and characterized, Furthermore, the enzyme was tested in vitro for its cytotoxicity, antitumor activity and assessed in vivo for its immunogenicity.

## Materials and methods

### Bacterial strain

*S. maltophilia* isolate which was previously recognized through a comprehensive screening program as a promising L-asparaginase producer. This strain was deposited under the code EMCC2297 at Microbiological Resources Centre (Cairo Mircen) and its 16 S rRNA sequence was submitted in NCBI database and deposited under the Accession code MG66599 (Abdelrazek et al. 2020). For short term preservation, Luria Bertani (LB) agar (Himedia, India) slants were used for the monthly sub-culture of single pure colonies of the test isolate while for long time preservation, the isolate was kept in 50% glycerol at -80 °C as stock.

### Induction for gene amplification using chloramphenicol

According to the method mentioned by Norgad et al., (Norgard et al. 1979) gene amplification was performed for *S. maltophilia* EMCC2297 isolate using different concentrations of chloramphenicol to enhance the productivity of the isolate, this experiment was done in triplicate. The increased chloramphenicol concentration was added to the resultant colonies from the last concentration in the scaled-up series. This was done by inoculating 50 ml sterile Brain Heart broth (BHB) (Himedia, India) supplemented with 50 µg/ml chloramphenicol in 250 ml Erlenmeyer flask with single colony of *S. maltophilia* EMCC2297 isolate obtained from overnight culture (Abdulrahman 2007). The flasks were incubated for 24 h at 37 °C and 180 rpm. The resultant broth was plated on Brain Heart agar (BHA) (Himedia, India) plates and the healthy colonies (showing the biggest size and healthiest appearance) were used as inoculum for the higher concentration of chloramphenicol. The selected colony was inoculated in 250 ml Erlenmeyer flask containing 100 µg/ml chloramphenicol and the same steps were performed for higher concentrations (200 & 300 µg/ml). The healthy colonies obtained from each concentration were quantitively screened for L-asparaginase production to detect the concentration that significantly increased the production of L-asparaginase. The obtained activity was statistically analyzed using one-way ANOVA followed by Tukey’s Multiple Comparison Tests.

### Purification of L-asparaginase produced by *S. maltophilia* EMCC2297

#### Inoculum preparation and L-asparaginase production

To prepare the inoculum, a single colony from the selected culture showing the highest L-asparaginase productivity among those subjected to gene amplification was inoculated into 20 ml of LB broth in a 250 ml Erlenmeyer flask. Then, the flask was incubated at 37 °C and 180 rpm for 24 h (Mahajan et al. 2012). The broth culture produced was diluted using fresh LB broth to reach an optical density (O.D.) value of 1.0 to be used as inoculum in the test. Erlenmeyer flask of 250 ml was used to produce the enzyme, an inoculum of 2% v/v from the cell suspension was then added to the flasks containing fresh double strength LB broth supplemented with 3% asparagine (AppliChem GmbH, Darmstadt, Germany). The flasks were incubated for 24 h at 37 °C and 180 rpm. Two ml aliquot of the obtained broth culture was centrifuged using cooling centrifuge for 20 min at 5000 rpm and 4 °C (Jain et al. 2012). The resultant supernatant was considered a crude enzyme preparation and was utilized for further purification process.

#### Enzyme purification

The purification was performed using the following steps: ammonium sulfate precipitation followed by size exclusion column chromatography (Sephadex G-100). For the ammonium sulfate precipitation, 1000 ml cell free culture supernatant of the test organism were prepared by centrifugation for 20 min at 5000 rpm and 4 °C. The protein contents of obtained supernatant were precipitated using 70% solid ammonium sulfate (472 gm/l) while stirring overnight at 4 °C (Desai and Hungund 2018). The precipitate was pelleted through centrifugation for 30 min at 18,000 rpm and 4 °C. The obtained pellets were dispersed in 0.05 M Tris-HCl buffer (pH 8.6). The prepared suspension was dialyzed in a dialysis tube with molecular weight cut-off 13,000Da against 250 ml of the same buffer with stirring at 4 °C overnight (Elleboudy et al. 2016). The dialyzed enzyme was subjected for further purification by loading into Sephadex G-100 size exclusion column (2.5* 30 cm). The column was pre-equilibrated using 0.05 M Tris HCl buffer pH 8.6 and L-asparaginase enzyme was eluted by the same buffer (Roy et al. 2019). One ml fractions were collected at a flow rate of 0.5 ml/min then tested for its protein content using Bradford assay at 25 °C with bovine serum albumin as a reference standard (Sigma-Aldrich Co., St. Louis, MO, USA) (Bradford 1976). Also, they were tested for L-asparaginase activity. The active fractions of L-asparaginase were pooled and used for further analysis (Elleboudy et al. 2016).

#### L-asparaginase activity assay

L-asparaginase activity was determined applying Mashburn and Wriston method (Ambreen et al. 2019). The principle of the method is that the resultant enzyme preparation was utilized to hydrolyze L-asparagine and release ammonia. The reaction mixture consisted of 0.5 ml of the enzyme preparation, 0.5 ml of 0.05 M Tris-HCl buffer (pH 8.6), and 0.5 ml of 0.04 M asparagine. The reaction mixture was then incubated for 30 min at 37 °C. The enzyme activity was stopped through the addition of 10% W/V TCA. The mixture was then centrifuged for 5 min at 10,000 rpm, afterwards 3.7 ml of distilled water was mixed with 0.1 ml of the resulting supernatant, followed by the addition of 0.2 ml Nessler’s reagent and the produced mixture was kept for 20 min at room temperature. The absorbance was spectrophotometrically measured at 480 nm. Ammonium sulphate standard curve was utilized to calculate the amount of liberated ammonia (Jain et al. 2012). One unit of L-asparaginase activity can be defined as the enzyme amount needed for the release of one micromole of ammonia per hour at pH 8.6 and 37 °C (Mahajan et al. 2014; Trang and Le Thanh Hoang 2018).

### Molecular weight determination of the purified native *S. maltophilia* L-asparaginase (N-SMASP)

Sodium Dodecyl Sulfate Polyacrylamide Gel Electrophoresis (SDS-PAGE) was carried out using a 15% polyacrylamide gel according to the method of Laemmli (Laemmli 1970). As a running buffer for electrophoresis, Tris-glycine buffer (pH 8.3) was applied which was operated at 90 V for 3 h at room temperature. To stain the proteins on the gel, Coomassie Brilliant Blue R-250 was used and a mixture of acetic acid, methanol, and water were used for destaining. Standard molecular weight marker was used to determine the apparent molecular weight of L-asparaginase.

### Kinetic parameters of the purified N-SMASP

The kinetic parameters for the purified enzyme were predicted using L-asparagine as a substrate at different concentrations in the range of 0.01–0.12 M which were included in the assay reaction mixture in 0.05 M Tris HCl buffer (pH 8.6). The assay was conducted at 37 °C and the obtained activities at the different enzyme concentrations were used for calculation of Michaelis constant (Km), peak velocity (Vmax), and turnover numbers (Kcat). By using the equation created from a non-linear regression analysis of the curve constructed between the asparagine concentration and the enzyme activity, the Km and Vmax were computed from Lineweaver-Burk plots. The equation Kcat = Vmax / [E], where [E] is the enzyme concentration employed in the reaction, was used to determine the Kcat value. Every reaction was carried out in triplicate (Husain et al. 2016).

### Evaluation of the cytotoxicity and antitumor activity of the purified N-SMASP in comparison to the commercial *E.coli* L-asparaginase

#### Cell lines and propagation

Three cell lines were utilized in this study [human hepatocellular cancer cell line (HepG-2) cells, human leukemia cancer cell line (K-562) cells, and normal human lung fibroblast cells (MRC-5)] and all were acquired from the American Type Culture Collection (ATCC, Rockville, MD). HepG-2 and K-562 cells were grown in Rosewell Park Memorial institute medium (RPMI-1640) (Sigma Aldrich, USA) supplemented with 50 µg/ml gentamycin and 10% inactivated fetal calf serum. The cells were kept at 37ºC in a humidified atmosphere with 5% CO_2_ and were sub-cultured two to three times a week (Cruz et al. 2020; dos Santos et al. 2020). On the other hand, MRC-5 cells were propagated in Dulbecco’s modified Eagle’s medium (DMEM) (Sigma Aldrich, USA) containing 10% heat-inactivated fetal bovine serum, HEPES buffer (4-(2-hydroxyethyl)-1-piperazineethanesulfonic acid), 1% L-glutamine, and 50 µg/ml gentamycin. All cells were kept at 37ºC in a humidified atmosphere with 5% CO_2_ and were sub-cultured two times a week (Hall et al. 2021).

#### Antitumor assay

Antitumor activity was evaluated for the purified native L-asparaginase enzyme and the commercial *E.coli* L-asparaginase (L-ASAP Neova, Biogene). The HepG-2 and K-562 cell lines were suspended in 96-well tissue culture plates (Corning®) containing the previously mentioned medium at a concentration of 5 × 10^4^ cell/well, then incubated for 24 h. The tested enzyme was then added into the plates (three replicates) to achieve twelve concentrations (six concentrations for each tumor cell) in the range of 0–7.276 IU/ml. Six controls with media or with 0.5% DMSO were run in parallel. After incubation for 48 h, the number of viable cells was predicted by the MTT (3-[4,5-dimethylthiazol-2-yl]-2,5 diphenyl tetrazolium bromide) test (Ghasemi et al. 2021). Briefly, the media were removed from the plate by gentle blotting on paper towel and replaced with 100 µl fresh culture RPMI 1640 medium without phenol red and 10 µl MTT stock solution of 12 mM (5 mg MTT in 1 mL of PBS) were added in each well including the controls. The plates were then incubated for 4 h at 37 °C and 5% CO_2_. For each well, the excess media were carefully removed by gentle blotting technique and 50 µl DMSO were added, mixed well using the pipette, and gently shaken using microtiter plate shaker to form a homogenous solution then the plate was incubated for 10 min at 37 °C. Then, using microplate reader (SunRise, TECAN, Inc, USA), the O.D. was measured at 590 nm to predict the number of viable cells. The viability percent was calculated using the following equation ([(O.D_t_/O.D_c_)]x100%), where (O.D_t_) is the mean optical density of wells treated with the tested enzyme, and (O.D_c_) is the mean optical density of untreated cells (Husain et al. 2016). For each tumor cell line, the survival curve after the treatment with each enzyme was obtained by plotting a relation between the surviving cells and the enzyme concentration. The 50% inhibitory concentration (IC_50_); the concentration required to cause toxic effects in 50% of intact cells; was calculated from the graphic plots of the dose response curve for each enzyme with the aid of Graphpad Prism software (San Diego, CA. USA) (Mosmann 1983; Riyadh et al. 2015; Tolosa et al. 2015).

### Cytotoxicity assay to human normal cell

To determine the safety of the N-SMASP , the cytotoxicity on normal human lung fibroblast cells (MRC-5 cells) was determined for the purified *S. maltophilia* EMCC2297 L-asparaginase in comparison to the commercial enzyme of *E.coli*. The MRC-5 cells were seeded in 96-well, flat-bottomed microtiter plates (Falcon, NJ, USA) at a concentration of 1 × 10^4^ cells in 100 µl growth medium per well. In a humidified incubator with 5% CO_2,_ the plates were incubated for 24 h at 37ºC. Two-fold serial dilutions of the tested L-asparaginase in fresh medium were added to the confluent cell monolayers obtained in the 96-well plates (about 24 h incubation period), utilizing a multichannel pipette. Under the same previously mentioned conditions, the microtiter plates were re-incubated for a period of 24 h. For each concentration of the test enzyme, three wells were used. Control wells were run in parallel without test sample and with or without DMSO. After incubation the percentage of viable cells were determined as mentioned before in antitumor assay (Gomha et al. 2015).

### Immunogenicity testing of N-SMASP and *E.coli* L-asparaginase

The JTT matrix-based model’s Maximum Likelihood method was previously used to determine the immunogenicity of *S. maltophilia*’s EMCC2297 L-asparaginase in comparison to those from *Erwinia chrysanthemi* and *E. coli* (marketed as Erwinaze and Elspar, respectively) (Abdelrazek et al. 2020). So, in vivo studies were performed to prove the results obtained from the bioinformatics analysis.

Male BALB/c mice of 6–8 weeks old weighing 20–30 gm were used to compare the immunogenicity of the native purified L-asparaginase and the commercially available L-asparaginase (L-ASAP Neova, Biogene). In open cages, animals were kept in an air-conditioned atmosphere at a temperature of 25 °C with alternating 12-hour cycles of light and dark at the animal house of the Faculty of Pharmacy (Future University in Egypt). They had unrestricted access to water and food free of antibiotics that contained at least 5% fiber, 20% protein, 6.5% ash, 3.5% fat, and a vitamin blend. Two groups of mice (*n* = 12/group) were injected intraperitoneal of either native purified *S. maltophilia* EMCC2297 L-asparaginase or the commercial L-asparaginase with a dose of 250 U/kg twice a week for 4 weeks (El-Naggar et al. 2018). A third group (*n* = 12) was assigned as the control group and received 50 mM Tris HCl buffer pH 8.6 (Rodrigues et al. 2020). ELISA method was used to determine the concentrations of specific antibodies against the two L-asparaginases in sera of the tested animals using anti-mouse anti-IgG Horse Radish Peroxidase. Phosphate buffered saline (PBS) containing 5% Skimmed milk and 0.05% Tween 20 was used to block the L-asparaginases-coated plates for 2 h at room temperature (McConnell et al. 2006). The plates were then thoroughly washed using PBS-Tween mixture and were incubated for 2 h with diluted serum samples. After that, the plates were washed, re-incubated for 1 h with the anti-mouse anti-IgG Horse Radish Peroxidase and washed again. The plates were incubated for 30 min with a 3, 3′, 5, 5′-tetramethylbenzidine substrate, and quenched using 9.8% (v/v) H_2_SO_4_ in water for the colour development. Fifty µl of the stop solution were added and the absorbance was estimated within 30 min at 450 nm by a plate reader. The value corresponding to the level of IgG antibody was obtained by subtracting the absorbance value of each sample from the blank value. The titre of IgG was calculated as the reciprocal of the highest dilution that had a reading higher than double the control value (Zenatti et al. 2018). The study received approval from the Research Ethics Committee of the Faculty of Pharmacy Ain Shams University, Egypt (ENREC – ASU 2020 − 101).

### Statistical data analyses and graphical representations

The experiments were conducted in triplicates, the mean values and standard deviation were then calculated, and the results were presented in graphs with error bars. The one-way ANOVA followed by Tukey’s Multiple Comparison Tests, Student t-test and two-way ANOVA were applied using GraphPad Prism 8 Software (GraphPad Inc., La Jolla, CA, USA) for data analyses.

## Results

### Induction for gene amplification using chloramphenicol

**Fig. 1:**
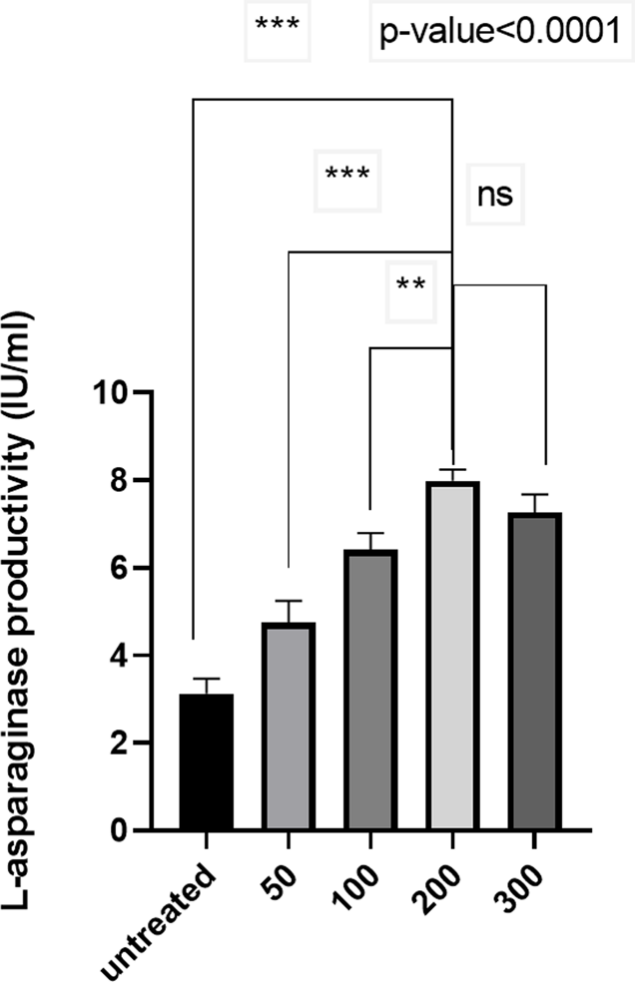
L-asparaginase production from the untreated *S. maltophilia* EMCC2297 and the selected colonies recovered after the exposure to different concentrations of chloramphenicol. The results represent the average of the data obtained from three independent experiments and the error bars indicate standard deviation. The results were statistically analyzed using one-way ANOVA followed by Tukey’s Multiple Comparison Tests. **, *p* value < 0.001; ***, *p* value < 0.0001

Different concentrations of chloramphenicol (50–300 µg/ml) were used to enhance the productivity of the *S. maltophilia* EMCC2297 isolate. The chloramphenicol concentration was increased gradually to the resultant colonies from each concentration. The colonies obtained from each concentration were tested for L-asparaginase productivity to determine the concentration that significantly increased the productivity of the enzyme. The results revealed that the recovered colonies from 200 µg/ml showed the highest L-asparaginase productivity. However, there was a significant gradual increase in L-asparaginase productivity when the cells were subjected to subsequent increasing concentrations of chloramphenicol of 50, 100 and 200 µg/ml as compared to the untreated cells (*p*-value < 0.0001). Further increase of chloramphenicol concentration to 300 µg/ml resulted in insignificant decrease in L-asparaginase productivity as compared to that obtained at 200 µg/ml concentration (Fig. 1). So, colonies recovered at 200 µg/ml chloramphenicol concentration were selected to complete the present study.

### Enzyme purification and molecular weight determination

**Fig. 2:**
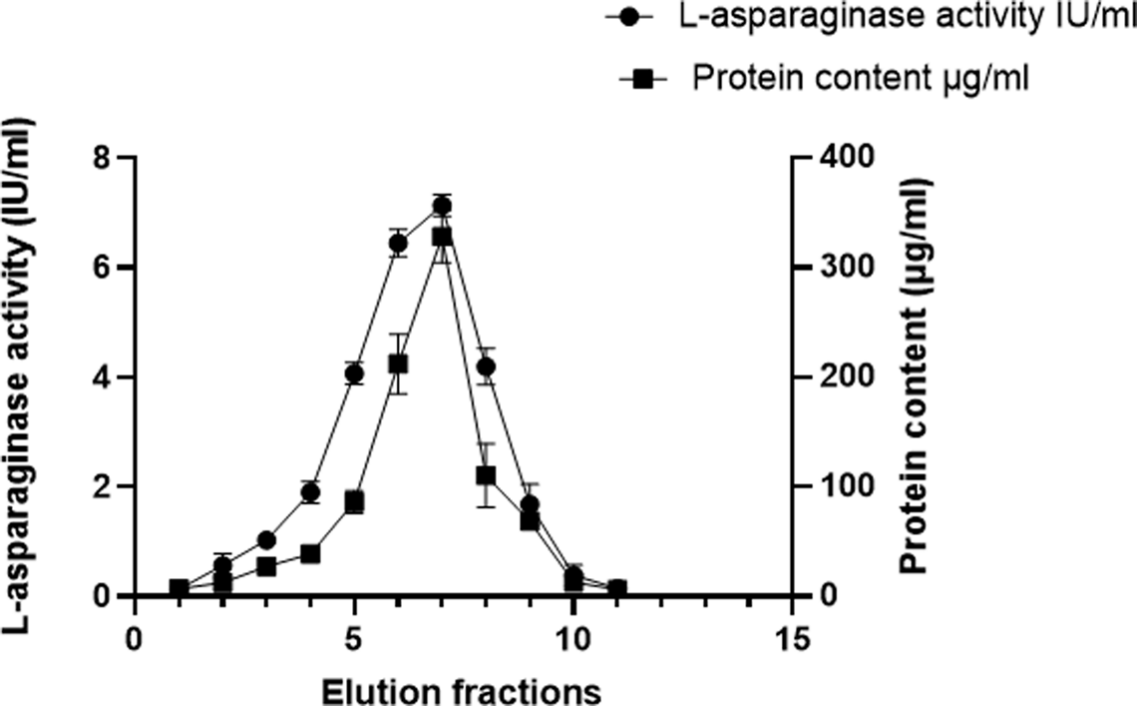
Gel-filtration of L-asparaginase from *S. maltophilia* EMCC2297. Elution was done with 0.05 M tris buffer (pH 8.6) at a flow rate of 0.5 ml/min and fraction volume of 4 ml. The results represent the average of the data obtained from three independent experiments and the error bars indicate standard deviation

The crude extract of *S. maltophilia* EMCC2297 isolate was partially purified by ammonium sulfate precipitation. The precipitate obtained were resuspended in 0.05 M Tris-HCl buffer (pH 8.6), dialyzed against 250 ml of the same buffer with stirring at 4 °C overnight, and subjected to gel filtration chromatography. Figure 2 shows the L-asparaginase activity and protein content for each elution fraction obtained from gel filtration chromatography. The fraction number 7 showed the highest enzyme activity as well as the highest protein content. The enzyme activity, specific enzyme activity and purification fold for the crude L-asparaginase preparation (crude extract), partially purified enzyme (obtained from ammonium sulfate precipitation) as well as the purified enzyme (obtained from gel filtration) are shown in Table 1. The purification folds of L-asparaginase obtained after gel filtration reached 5.48 folds while the yield was 30% of the purified enzyme.

**Fig. 3:**
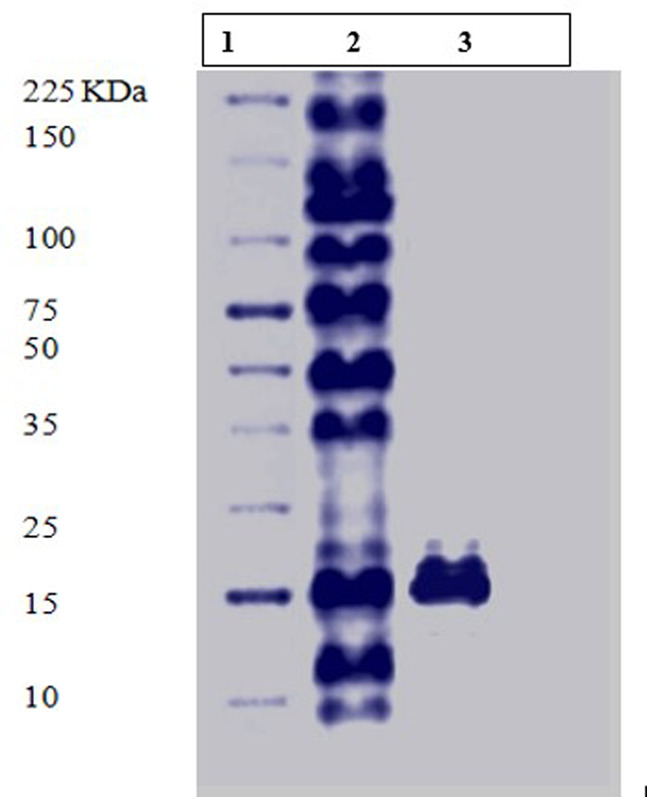
SDS-PAGE of the purified L-asparaginase from *S. maltophilia* EMCC2297. Lane 1: protein marker; Lane 2: crude extract; Lane 3: purified protein obtained after gel filtration

SDS-PAGE were performed to assess the molecular weight and the purity of the native purified L-asparaginase. The result showed that the obtained enzyme preparation was homogenously purified as it apeared on SDS-PAGE as a single band with an apparent molecular weight of 17 KDa (Fig. 3).

**Table 1:** Purification of L-asparaginase obtained from *S. maltophilia* EMCC2297 isolate

Purification steps	Total activity* (IU)	Protein content* (mg)	Specific activity (IU/mg)	Purification folds	Yield %
Crude extract	11,648	1745.812	6.671	1	100
Ammonium sulfate precipitation	5744	617.2705	9.305	1.409	49
Sephadex G100	3496	96.4375	36.251	5.484	30

* This represented the total activity/protein content recovered from 500 ml of culture

### Kinetic parameters of the purified N-SMASP

**Fig. 4:**
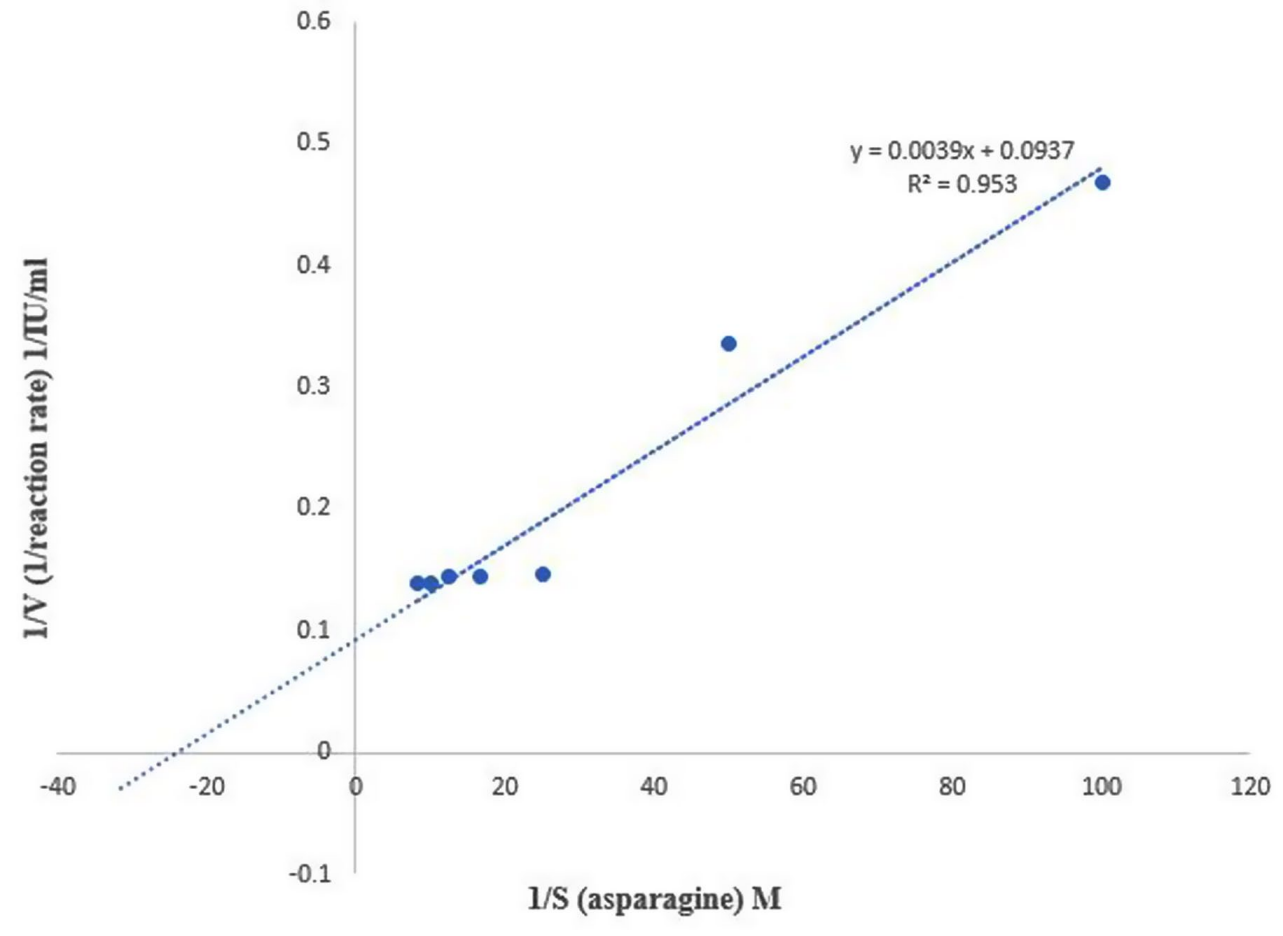
The lineweaver burk plot of *S. maltophilia* EMCC2297 L-asparaginase for detecting the kinetic parameters

The kinetic parameters of the purified L- asparaginase were determined by generating the Lineweaver- Burk plot with enzyme activity values measured at the optimal pH and temperature as shown in Fig. 4. The km was 4.16 × 10^− 2^ M, Vmax 10.67 IU/ml while the kcat was 1.466 S^− 1^.

### Evaluation of the antitumor activity and cytotoxicity of the purified N-SMASP using the viability assay

**Fig. 5:**
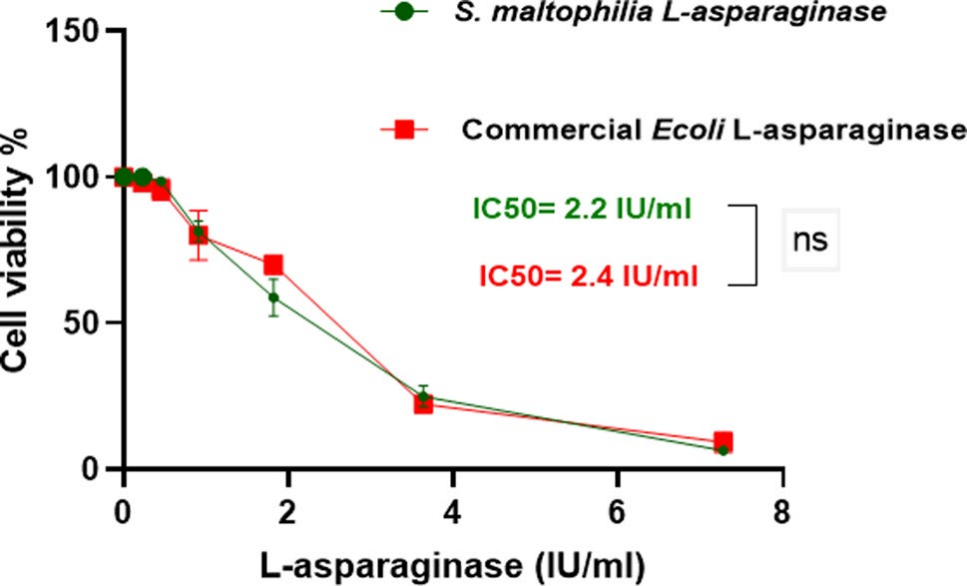
Antitumor activity of *S. maltophilia* EMCC2297 purified L-asparaginase and the commercial *E.coli.* on HepG-2 cells. The results represent the average of the data obtained from three independent experiments and the error bars indicate standard deviation. The results were statistically analyzed using Student t-test and there was no significant difference between the two enzyme sources

**Fig. 6:**
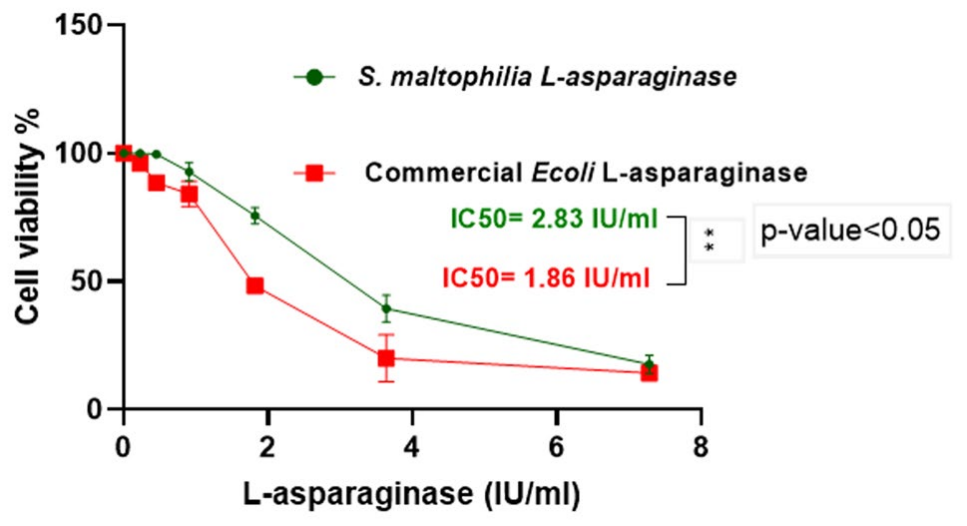
Antitumor activity of *S. maltophilia* EMCC2297 purified L-asparaginase and the commercial *E. coli* on K-562 cells. The results represent the average of the data obtained from three independent experiments and the error bars indicate standard deviation. The results were statistically analyzed using Student t-test and there was significant difference (*p*-value < 0.05) between the two enzyme sources

**Fig. 7:**
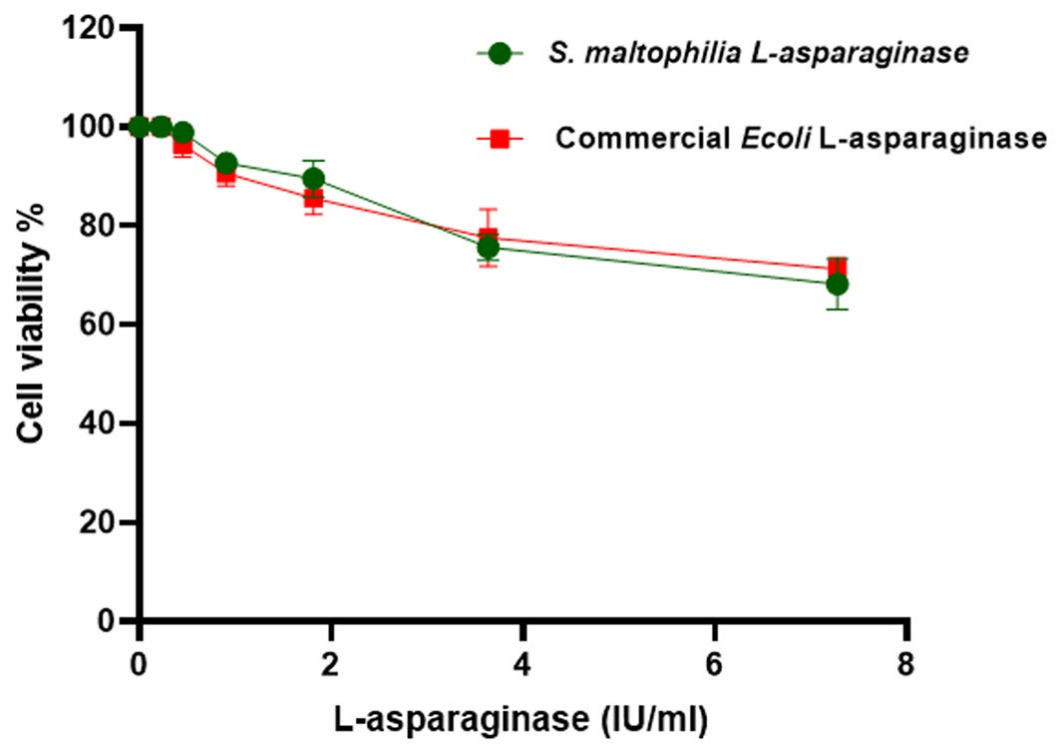
Cytotoxic activity of *S. maltophilia* EMCC2297 purified L-asparaginase and the commercial *E. coli* on MRC-5 cells. The results represent the average of the data obtained from three independent experiments and the error bars indicate standard deviation

The antitumor activity of the purified *S. maltophilia* EMCC2297 L-asparaginase and the commercial *E. coli* L-asparaginase were assessed using the MTT assay. The HepG-2 and K-562 tumor cells were effectively responded to the purified L-asparaginase at low doses, as 75% and 60% of HepG-2 and K-562 cells were killed using the purified *S. maltophilia* EMCC2297 L-asparaginase at a concentration of 3.6 IU/ml, respectively. The results revealed that incubating the cells with increasing doses of L-asparaginases were led to dose-dependent cytotoxicity (Figs. 5 and 6). IC50 of the purified L-asparaginase against HepG-2 was less than that of the commercial one, where IC50 for the purified and commercial L-asparaginase was found to be 2.2 IU/ml and 2.4 IU/ml, respectively, and these results showed non-significant difference to each other. While for K-562 cells there was a significant difference between IC50s of the purified enzyme compared to that of the commercial one, where IC50s were 2.83 IU/ml and 1.86 IU/ml for the purified enzyme and the commercial one, respectively. Moreover, *S. maltophilia* EMCC2297 L-asparaginase was found to be non-cytotoxic for normal cells MRC-5 as the same as the commercial *E.coli* as shown in Fig. 7.

### Immunogenicity testing

**Fig. 8:**
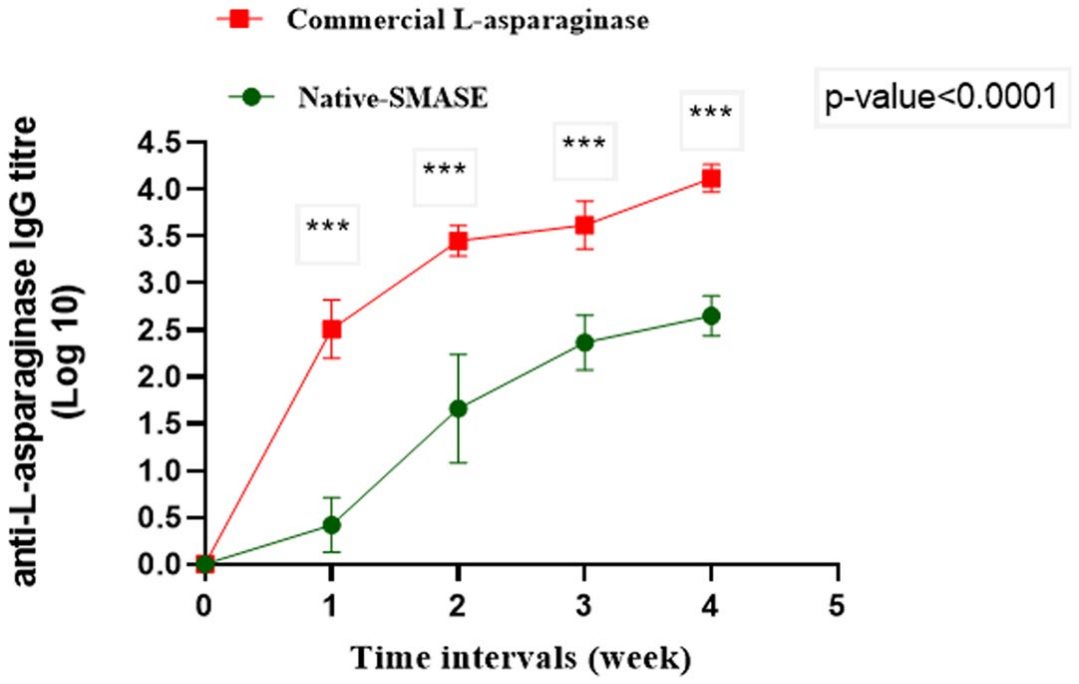
Development of anti-L-asparaginase IgG in terms of log_10_ titre over time in mice treated with the N-SMASP EMCC2297 and the commercial *E. coli* L-asparaginases. The results represent the average of the data obtained from three independent experiments and the error bars indicate standard deviation. The results were statistically analyzed using Two-way ANOVA Tukey’s Multiple Comparison Test. The results showed significant difference in the immunogenicity of tested L-asparaginases from the two sources (*p*-value < 0.0001)

The immunogenicity profile of the purified N-SMASP and the commercial one from *E. coli* in mice was characterized. The levels of IgG against both L-asparaginases were evaluated in sera of treated animals using ELISA method. Both N-SMASP and the commercial *E. coli* L-asparaginase were injected intraperitoneal in two mice groups and anti L-asparaginase IgG levels were measured in the collected sera of treated animals at different time intervals. The results showed gradual increase in the titre of anti-L-asparaginase IgG for both administered L-asparaginases as shown in Fig. 8. The results revealed that *S. maltophilia* EMCC2297 L-asparaginase is less immunogenic than that of the commerically available L-asparaginase with a significant difference (*p* < 0.0001) in the IgG titre levels. These results assured our previuosly determined bioinformatics results indicating that *S. maltophilia* EMCC2297 L-asparaginase had low immunogenicity as compared to the *E. coli* originated one (Abdelrazek et al. 2020).

## Discussion

Microbial L-asparaginase has been widely applied because of its potential as an anticancer agent (Ahmad et al. 2012; Cachumba et al. 2016). Several investigations have revealed that L-asparaginase serves as an anti-tumor agent due to its high efficiency in converting asparagine (found in abnormally high proportions in malignant cells) into aspartic acid and ammonia (Golbabaie et al. 2020; Kumar et al. 2022). As a result, L-asparaginase is a necessary tool in the treatment of acute lymphoid system and melanosarcoma cancers (Mukherjee 2019; Wang et al. 2018). L-asparaginase suppresses cancer cell proliferation by depleting the non-essential amino acid L-asparagine, which is a crucial nutrition for tumor cells.

Previous Bioinformatic analysis revealed that the L- asparaginase of *S. maltophilia* EMCC2297, the target for production and purification in this study, exhibits lower immunogenicity when compared to the commercial counterpart from *E.coli* and *Erwinia chrysanthemi* (Abdelrazek et al. 2020). This study aimed at the production and purification of a promising *S. maltophilia* EMCC2297 L-asparaginase in a simple and cost-efficient way. The purified enzyme was assessed for its catalytic properties, antitumor activity, cytotoxicity, and immunogenicity in comparison to the commercially available L-asparaginase.

Researchers have investigated the use of chloramphenicol in the induction of certain gene amplification which may aid in increasing the productivity of certain enzymes in bacteria and human cells. *S. maltophilia* is naturally resistant to a wide range of antibiotics including chloramphenicol (Bray et al. 2020; de Prevenção 2019). This resistance is due to the presence of genes encoding efflux pumps and antibiotic inactivating enzymes on its chromosome (Bray et al. 2020; de Freitas Rodrigues et al. 2023; Sung et al. 2021). The degree of amplification can be adjusted by adjusting the concentration of chloramphenicol in the growing medium (Berridge et al. 2005). In this study gene amplification induction was tested for L-asparaginase production from chloramphenicol resistant *S. maltophilia* EMCC2297. The organism was exposed subsequently for increased concentrations of chloramphenicol to gradually increase the stress on the bacterial cell and to force it to amplify certain genes in its genome. This was followed by L-asparaginase quantitative assay to the healthy surviving cells. The results revealed that by increasing the chloramphenicol stress on the cells, there was a significant increase in L-asparaginase production till 200 µg/ml chloramphenicol concentration. Further increase in the concentration to 300 µg/ml showed no significant difference in the productivity when compared with that at 200 µg/ml. So, the 200 µg/ml chloramphenicol survival cells were chosen to complete the present study. Our results were in agreement with many researches that concluded that chloramphenicol could successfully amplify bacterial DNA and enzyme productivity (Abdulrahman 2007; Berridge et al. 2005; Ferrari et al. 1990; Niles et al. 2007).

L-asparaginase enzyme typically coexists with other proteins, nucleic acids, polysaccharides, and lipids. The enzyme can be purified and its specific activity can be increased by removing contaminated material using a variety of techniques. In this study, L-asparaginase of *S. maltophilia* EMCC2297 isolate was purified by utilizing gel filtration chromatography on Sephadex G-100 column after ammonium sulphate precipitation and dialysis. Gel filtration is also called molecular sieve chromatography or size exclusion chromatography in which the separation is based on differing ability of molecules in the sample (due to different molecular sizes) to enter the pores of the gel filtration media (Ghasemi et al. 2021). L-asparaginase was purified up to 5.48 folds with specific activity of 36.251 IU/mg protein, Hussien et al. (2016) purified L-asparaginase from *Enterobacter cloacae* by ammonium sulfate precipitation, ion exchange chromatography and gel exclusion chromatography to 105 IU/mg protein and 119 folds increase enzyme activity (Husain et al. 2016). While Rahimzadeh et al. (2016) and Naggar et al., (2018) purified intracellular and extracellular L-asparaginase from *Bacillus* PG03, *Bacillus* PG04 and *Streptomyces brollosae* by sonication of the cell followed by anion exchange chromatography using DEAE Sepharose column. The results of the purified L-asparaginase from *Bacillus* PG03 and *Bacillus* PG04 were 1.2 mmole/min activity and 7.8 fold purification with 69 IU/mg protein (Rahimzadeh et al. 2016). Likewise Parashiva et al., (2023) purified L-asparaginase from *Fusarium foetens* by ammonium sulfate fractionation, dialysis and ion exchange chromatography using DEAE cellulose column with specific activity of 231 IU/mg protein and 15.6 fold purification (MAZLUMOĞLU 2023).

The single band observation on SDS-PAGE confirmed homogenous purification of L-asparaginase from *S. maltophilia* EMCC2297. The purified L-asparaginase molecular mass was estimated to be 17 KDa. Different molecular weight was estimated for L-asparaginase from different microorganisms as it was purified from *Streptomyces brollosae*, *Fusarium foetens, Enterobacter cloacae* and *Streptomyces fradiae* with molecular weights of 67 KDa, 37 KDa, 52 KDa, 53 KDa, respectively (El-Naggar et al. 2016; Husain et al. 2016). Moreover a 25 KDa and 30 KDa L-asparaginases were purified from *Bacillus* PG03, *Bacillus* PG04, respectively (Rahimzadeh et al. 2016).

The Michaelis-Menten model of enzyme kinetics explains how the concentration of an enzyme and its substrate affects the rate of an enzyme-catalyzed reaction (Da Costa et al. 1999). Two important terms within Michaelis-Menten kinetics, the first is the Vmax which is defined as the reaction maximum rate, when the substrate saturates all the enzyme’s active sites. The second is the Km (also called the Michaelis constant) which is the concentration of the substrate at half maximum reaction rate (Vmax/2). Km is a measure of the enzyme affinity for its substrate; the lower the value of Km, the more efficient the enzyme is at carrying out its function (Da Costa et al. 1999). Also, a low Km value means that the enzyme can get saturated with a relatively small amount of substrate. Therefore, relatively low substrate concentrations are where the highest velocity is obtained. A high Km value means that high substrate concentrations are required to obtain the fastest reaction rate (Robinson 2015).

Investigating the kinetic parameters of L-asparaginase purified from *S. maltophilia* EMCC2297 revealed that Vmax was 10.67 IU/µg. The turnover numbers (kcat) or the catalytic rate constant which is the maximal number of substrate molecules converted to product per active site per unit time for several different substrates to different products was 1.466 S^-1^. While the km was 4.16 × 10^− 2^ M which is relatively the same as that detected for purified L-asparaginase from *Bacillus licheniformis* 0.049 M (Alrumman et al. 2019). The low value of Km obtained from purified L-asparaginase of *S. maltophilia* EMCC2297, denotes the enzyme’s strong affinity for the substrate L-asparagine and is necessary for the targeted elimination of L-asparagine in case of leukemia cells (Sharma and Mishra 2023a). Earlier studies for L-asparaginase kinetic parameters had revealed different Km values from different microorganisms which was 0.059 mM for that produced from *Pseudomonas* sp. PCH44 (Kumar et al. 2022), 1.58 × 10^− 3^ M for that of *Enterobacter cloacae* (Husain et al. 2016) and 9.74 mM for that of *Sarocladium strictum* (Golbabaie et al. 2020).

The *in-vitro* antitumor effect of purified L-asparaginase isolated from *S. maltophilia* EMCC2297 and the commercial one was tested on the most widespread and significant severe type of cancer (leukemic cell K-562 and liver cancer cell Hep-G2) using MTT assay. The antitumor potential was evaluated in terms of IC50, the concentration dose necessary to eradicate 50% of cancerous cells following a 48-hour incubation period. The results showed IC50 for the purified L-asparaginase of 2.83 IU/ml for K-562 and 2.2 IU/ml for Hep-G2. While the commercial L-asparaginase showed IC50 of 1.86 IU/ml for K-562 and 2.4 IU/ml for Hep-G2. The low value of IC50 is indicative for potential drug action and so lesser systemic toxicity when given to patients since a medicine is more effective at low doses (Berrouet et al. 2020). The increase in L-asparaginase concentration was associated with an increase in the cytotoxicity against malignant cells, indicating that the cytotoxicity was dose-dependent (Sharma and Mishra 2023b). Because the deamination of the non-essential amino acid asparagine resulted in a decrease in asparagine pool, the anticancer activity of L-asparaginase revealed the effective death of malignant cell types (Darnal et al. 2023). These results indicate that purified L-asparaginase has no significant difference compared with the commercial one when tested on Hep-G2 cells while its effect on K-562 was significantly different. Many researches had supported the effectiveness of L-asparaginase against leukemic cells, as L-asparaginase isolated from *Melioribacter roseus* has nearly the same IC50 (3.0 IU/ml) (Sivakumar et al. 2023) of *S. maltophilia* EMCC2297 L-asparaginase. Various L-asparaginases have different IC50 as the one isolated from *Rhodospirillum rubrum* has IC50 1.8 IU/ml (Dobryakova et al. 2023) and of *Halomonas elongate* has IC50 2.0 IU/ml (Ghasemi et al. 2017) also L-asparaginase isolated from *E.cloacae* has reported IC50 equals 7.1 IU/ml (Husain et al. 2016). Other researchers had supported the effectiveness of L-asparaginase on liver cancer Hep-G2 cells as the isolated ones from *Streptomyces rochei* and *Bacillus velezensis* had a cytotoxic activity against Hep-G2 (El-Naggar and El-Shweihy 2020; Mostafa et al. 2019) and also reported high IC50 equals 4.0 IU with *Streptomyces fradiae* NEAE-82 (El-Naggar et al. 2016). To ascertain the enzyme selective toxicity against tumor cells and minimal cytotoxic effect on normal healthy cells , the purified L-asparaginase from *S. maltophilia* EMCC2297 has been tested on normal cell MRC-5. Fortunately, it showed no cytotoxic activity on the normal cells. This may be attributable to asparagine synthetase activity, which utilizes substrate supplied by other activities during depletion (Darnal et al. 2023).

The basic barrier limiting the use of foreign proteins in human medicine is the immunogenicity of the drug (El-Naggar et al. 2018). Real immunological tolerance, which calls for antigen-specific T-cell-mediated immunosuppression, is challenging to acquire. Changing to a different preparation is one approach to temporarily solve this problem. In our previous study, *E. coli*, *Erwinia chrysanthemi*, and *S. maltophilia* EMCC2297 L-asparaginases antigenic regions discovered by EMBOSS antigenic explorer® analysis (Rice et al. 2000) were 18, 16, and 14 antigenic regions, respectively (Abdelrazek et al. 2020). The examination of the antigenic epitopes revealed that *S. maltophilia* EMCC2297 L-asparaginase had the lowest antigenicity, followed by *Erwinia chrysanthemi*, whereas *E. coli* had the maximum number of antigenic regions. According to Cavanna et al. (1976a) partial immune mediated negative effects of these enzymes are present when L-asparaginases from *Erwinia* and *E. coli* are used medically. This makes it necessary to look for alternative microbial L-asparaginases with less immune-mediated adverse effects. The low antigenic epitopes that have been observed in *S. maltophilia* EMCC2297 L-asparaginase may indicate fewer side effects and could warrant further experimental testing using an *in*-*vivo* animal model to demonstrate the possibility of using the enzyme as a therapeutic candidate for pharmaceutical and medical applications.

In this study, male BALB/c mice of 6–8 weeks old weighing 20–30 gm were used to compare the antigenicity effects of the purified L-asparaginase and the commercially available one. Young mice were chosen because they exhibit higher immunological responses than older animals (Stark et al. 2019; Stiasny et al. 2012). The sensitive and robust immune system of BALB/C mice is well known. They have excellent T-cell function and cytokine releasing capacity (Jiskoot et al. 2016). To assess the bioinformatics predicted low antigenicity *S. maltophilia* EMCC2297 L-asparaginase, the mice were injected intraperitoneally with either purified *S. maltophilia* EMCC2297 L-asparaginase or the commercial *E. coli* L-asparaginase. The ELISA method was used to determine the concentrations of IgG antibodies against the purified L-asparaginase and the commercial L-asparaginase in collected animal sera. It was confirmed that the native *S. maltophilia* EMCC2297 L-asparaginase is less immunogenic than the commercial one with a significant difference (*p* < 0.0001) in IgG titre levels. So, the current investigations provide evidence for the expected low antigenicity of the isolated *S. maltophilia* EMCC2297 L-asparaginase upon its introduction in medical application and supports it as a potential candidate for cancer treatment.

In conclusion, this study reveals that *S. maltophilia* EMCC2297 L-asparaginase can be optimized for higher production at low cost. It also discloses its potential anticancer activity with higher selective toxicity and less immunogenicity when compared to commercial *E.coli* L-asparaginase. Hence, *S. maltophilia* EMCC2297 L-asparaginase could be considered as a promising candidate for medical use as antitumor agent.

## Data Availability

Please contact author for data request.

## Abbreviations

RPMI: Rosewell Park Memorial institute medium
HEPES buffer: 4-(2-hydroxyethyl)-1-piperazineethanesulfonic acid
DMEM: Dulbecco’s modified Eagle’s medium
MTT: 3-[4,5-dimethylthiazol-2-yl]-2,5 diphenyl tetrazolium bromide
PBS: Phosphate buffer saline
DMSO: Dimethyl sulfoxide
IC_50_: Half maximal inhibitory concentration
