# Correction to: Production of highly cytotoxic and low immunogenic L-asparaginase from *Stenotrophomonas maltophilia* EMCC2297

**DOI:** 10.1186/s13568-025-01848-y

**Published:** 2025-03-29

**Authors:** Nada A. Abdelrazek, Sarra E. Saleh, Marwa M. Raafat, Amal E. Ali, Mohammad M. Aboulwafa

**Affiliations:** 1 Department of Microbiology and Immunology, Faculty of Pharmacy, Future University in Egypt, Cairo, Egypt; 2 Department of Microbiology and Immunology, Faculty of Pharmacy, Ain Shams University, African Union Organization St. Abbassia, Cairo, 11566 Egypt; 3 Department of Microbiology and Immunology, Faculty of Pharmacy, King Salman International University, Ras Sudr, South Sinai, Egypt

**Correction to: AMB Express (2024) 14:51** 10.1186/s13568-024-01700-9

The original publication of this article (Abdelrazek et al. 2024) contained incorrect affiliations for the authors. The correct and incorrect affiliations are listed in this correction article.

Correct Affiliations:

^1^Department of Microbiology and Immunology, Faculty of Pharmacy, Future University in Egypt, Cairo, Egypt

^2^Department of Microbiology and Immunology, Faculty of Pharmacy, Ain Shams University, African Union Organization St. Abbassia, Cairo, 11566, Egypt

^3^Department of Microbiology and Immunology, Faculty of Pharmacy, King Salman International University, Ras Sudr, South Sinai, Egypt

Incorrect Affiliations:

^1^Department of Microbiology and Immunology, Faculty of Pharmacy, Ain Shams University, Al Khalifa Al Maamoun St., Abbassia, Cairo, 11517, Egypt

^2^Department of Microbiology and immunology, Faculty of Pharmacy, Future University in Egypt, Cairo, Egypt

^3^Department of Microbiology and Immunology, Faculty of Pharmacy, King Salman International University, South Sinai, Ras-Sudr, Egypt
